# Current Knowledge on Infectious Bronchitis Virus Non-structural Proteins: The Bearer for Achieving Immune Evasion Function

**DOI:** 10.3389/fvets.2022.820625

**Published:** 2022-04-08

**Authors:** Shuwei Peng, Yiming Wang, Yu Zhang, Xu Song, Yuanfeng Zou, Lixia Li, Xinghong Zhao, Zhongqiong Yin

**Affiliations:** Natural Medicine Research Center, College of Veterinary Medicine, Sichuan Agricultural University, Chengdu, China

**Keywords:** infectious bronchitis virus, non-structural proteins, accessory proteins, innate immune response, immune evasion

## Abstract

Infectious bronchitis virus (IBV) is the first coronavirus discovered in the world, which is also the prototype of gamma-coronaviruses. Nowadays, IBV is widespread all over the world and has become one of the causative agent causing severe economic losses in poultry industry. Generally, it is believed that the viral replication and immune evasion functions of IBV were modulated by non-structural and accessory proteins, which were also considered as the causes for its pathogenicity. In this study, we summarized the current knowledge about the immune evasion functions of IBV non-structural and accessory proteins. Some non-structural proteins such as nsp2, nsp3, and nsp15 have been shown to antagonize the host innate immune response. Also, nsp7 and nsp16 can block the antigen presentation to inhibit the adapted immune response. In addition, nsp13, nsp14, and nsp16 are participating in the formation of viral mRNA cap to limit the recognition by innate immune system. In conclusion, it is of vital importance to understand the immune evasion functions of IBV non-structural and accessory proteins, which could help us to further explore the pathogenesis of IBV and provide new horizons for the prevention and treatment of IBV in the future.

## Introduction

Infectious bronchitis virus (IBV) is the prototype of gamma-coronavirus belonging to coronaviridae family, order Nidovirales, which is the first coronavirus discovered in the world as early as 1930s (1, 2). Nowadays, IBV has been currently widespread worldwide. After infected with IBV, in addition to causing respiratory diseases, it also has multiple tissue tropisms such as the kidney, gastrointestinal system, and reproductive system, causing pathological damage to multiple organs in chicken, which seriously endangered the poultry industry and caused huge economic losses (3–5). Since IBV is a kind of positive-strand RNA virus, its proof-reading activity in replication is not strict as other DNA viruses, which makes IBV prone to mutation (2, 6–8). It has also resulted that IBV has many genotypes and serotypes in poultry industry, which is difficult for conventional vaccination to achieve a good protective effect (9, 10).

**Figure 1:**
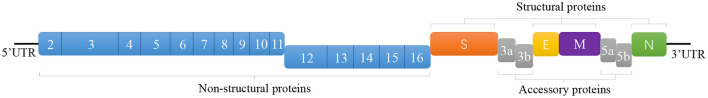
The infectious bronchitis virus genome structure from 5′ to 3′ UTR.

The total length of the IBV genome RNA is about 27.6 Kb (1), which sequentially encodes 5′-UTR, replicase gene 1a, replicase gene 1b, spike, gene 3a/b, envelope, membrane, gene 5a/b, nuclecapsid, and 3′-UTR (8, 11) (shown as Figure 1). Among them, the replicase genes 1a and 1ab occupy two-thirds of the entire genome length, encoding the polyprotein pp1a and pp1ab, which are then cleaved into 15 non-structural proteins (nsp2 to nsp16) under the hydrolysis of papain-like protease and 3C-like protease (9, 12). The non-structural proteins can play important roles in viral replication and regulation of host immune responses, and they are also considered as the main causes of IBV pathogenicity (4). In addition, IBV also encodes two accessory genes, gene 3 and gene 5, which are missing in some IBV strains (2). Accessory proteins were originally thought to be unnecessary during virus replication, but recent studies have found that they may be closely related to the pathogenicity of IBV (4).

After been infected with IBV, the innate immune response will be activated in chicken. First of all, the pathogen pattern recognition receptors (PRRs) of host cells can recognize the viral components, and thereby they can rapidly activate the innate immune response to inhibit the invasion and replication of IBV through a variety of pathways (13). Next, through Toll-like receptors on the cell membrane and MDA5 receptors located in the cytoplasm, NF-kB/IRFs, and MAPK signal pathways can be activated to promote the expression of inflammatory cytokines and interferons downstream of these pathways (14–17). However, with a long-term evolution and adaptation of the virus and the host, the virus has evolved a series of mechanisms to evade the host innate immunity, including blocking the host innate immune response or making it insufficient to inhibit the replication and pathological damage (17), and even causing immunosuppression of organs (5), which are conducive to the proliferation of viruses in host cells.

It is precisely because of its rich immune evasion function that IBV can limit the defense mechanisms of host cells, so as to achieve replication in host cells and even cause severe pathological damage. The immune evasion function of IBV is regulated by its non-structural proteins and accessory proteins (2). In view of the current difficulties for controlling IBV in the poultry industry through vaccines, it is necessary to understand the detail function of IBV proteins and the mechanisms involved in immune evasion, which can provide new targets to control IBV. In this review, from the perspective of IBV non-structural proteins, we reviewed the current knowledge of IBV non-structural proteins in recent years, and summarized the research frontiers of IBV immune evasion mechanism, which aimed to explore new horizons for the prevention and treatment of IBV.

## Non-Structural Proteins

### Nsp2: Antagonize the Activity of PKR

Compared with other coronaviruses encoding both nsp1 and nsp2, the non-structural protein encoded by IBV lacks nsp1 naturally, and therefore nsp2 becomes the first protein expressed in the IBV genome (18, 19). Initially, it is predicted that nsp2 is essential for IBV infection and may be involved in suppressing the host innate immune response (18). The alpha subunit of eukaryotic initiation factor 2 (eIF-2α) is a regulatory subunit of eIF-2, which can play a negative regulatory role on protein synthesis (20). Generally, after infected with viruses, the eIF2α kinase 2, also known as PKR, will be activated by viral dsRNA (21). The phosphorylated PKR will regulate the phosphorylation of eIF-2α, resulting in the stagnation of protein synthesis to let cells enter a defense state (22). A study found that after infected with IBV, the phosphorylation level of PKR and eIF-2α were significantly reduced, thereby maintaining the synthesis of proteins in the infected cells (23). Then, the structural and non-structural proteins of IBV were overexpressed, respectively, and it was found that nsp2 has weaker PKR antagonistic activity, which indicated that nsp2 may participate in the regulation of host immune response during IBV infection and promote the replication of IBV (23).

### Nsp3: Block the Host Innate Immune Response

Nsp3 is located at nucleotide positions 2548–8865 in the open reading frame (ORF1) of the IBV genome (24), which is also the largest non-structural protein encoded in the IBV genome. Among them, the papain-like protease domain encoded in nsp3 (nucleotides 4243–5553) (25) has multiple functions. Firstly, it can act as a protease to cleave the polyprotein PP1a and release the mature viral non-structural proteins (nsp2, nsp3, and nsp4) (26, 27). In addition, more importantly, the papain-like protease has strong deubiquitinating and deISGylating (Interferon Stimulated Genes, ISG) activity (28), which can remove the long polyubiquitin chain linked by Lys-K48 and Lys-K63 (26, 29). Therefore, IBV papain-like protease can remove the ubiquitin modification of proteins in antiviral innate immune pathways, thereby blocking or delaying the host innate immune response in IBV-infected cells (26, 28). It is an important immune evasion mechanism of IBV, which plays an extremely critical role for the replication of IBV in host cells (26, 28, 30). There is a research (27) showed that IBV papain-like protease can inhibit the production of IFN-β in DF-1 cells infected with IBV. Next, it was found that in the innate immune signal pathways which induce IFN-β expression, the ubiquitin modification levels of MDA5, TBK1, and their linker molecules MAVS, IKKε, and IRF3 were also reduced (27). The ubiquitination of these proteins is essential for the activation of signaling pathways, and the deubiquitination induced by papain-like protease prevents these proteins from participating in signal transduction, thus blocking the antiviral signaling pathways (27). At present, it has proved that in the innate immune signaling pathways, including NF-κB, RIG-I, and IFN stimulating factors, the signal transduction relies on the modification of polyubiquitin chains on specific proteins (29) and IBV papain-like protease can widely block the activation of these pathways through its deubiquitinating activity. In addition, other studies on papain-like protease have shown that it can not only suppress the host immune response, but also regulate other aspects of host cell life, such as the cell cycle (31), which suggested that nsp3 papain-like protease may also has other regulatory effects on host cells to promote IBV replication. Phillips et al. reported a comparative study on the complete gene sequence of pathogenic and attenuated IBV strains of the same serotype, and found that 34.75–43.66% of amino acid differences occurred in nsp3, which was much higher than the spike protein (5.8–13.4%) that was considered to be the largest difference. This result indicated that nsp3 may play an important role in IBV replication and virulence (32).

### Nsp4: Induce the Membrane Rearrangement

After infecting cells, viruses need to create the necessary environment and conditions for their own replication. Reorganization of cellular membranes is one of the important links. IBV has been shown to induce the synthesis of double membrane vesicles (DMVs), zipper endoplasmic reticulum (zER), and tethered vesicles (spherules) in infected cells (33, 34). It was found that IBV nsp4 can independently induce membrane rearrangement, provide a site for the assembly and synthesis of viral RNA, and protect it from the host antiviral immune recognition (33). However, it should be noted that although nsp4 can play a necessary role in the process of membrane rearrangement, it still needs to cooperate with viral or host factors to realize the whole process of IBV replication and assembly (34).

### Nsp6: Promote the Formation but Limit the Expansion of Autophagosomes

A screening and verification experiment on IBV non-structural proteins found that IBV nsp6 can activate autophagy (35). However, autophagy has a dual effect on viral replication process. On the one hand, the activation of autophagy pathways in host cells can trigger the degradation of viral components, and this is actually an innate immune defense response of host cells, which will not be conducive for viral replication (36). On the other hand, autophagosomes can promote the assembly of viral replicase protease to promote infection (37, 38). For this contradictory relationship, another study (36) confirmed that IBV nsp6 can limit the expansion of autophagosomes and omegasomes, causing them to lose their function of delivering viral components to lysosomes for degradation. All in all, IBV nsp6 can promote the formation of autophagosomes in the endoplasmic reticulum, which is conducive to its own component assembly process. At the same time, it can also limit the expansion and control the size of autophagosomes to avoid the adverse effects of autophagy for viral replication.

### Nsp7 and Nsp16: Block the Antigen Presentation

Generally, it is believed that the nsp7/nsp8 complex is used as a primer enzyme to bind with IBV nsp12 and play a vital auxiliary role in the synthesis of viral RNA (39–41). Recently, there was a report showing that IBV nsp7 and nsp16 can inhibit the maturation and cytokine secretion of bone marrow dendritic-like cells, and then block its antigen presentation ability (42). It implied that in addition to antagonizing the innate immune process, the non-structural proteins of IBV may also participate in the regulation of specific immune responses in the late stage of infection.

### Nsp13: Block the Cell Cycle

Nsp13 is also an important enzyme in the IBV replication process, which is usually called as helicase (41). Nsp13 is a multifunctional protein with a zinc-binding domain at the N-terminus and a helicase domain at the C-terminus (41). Fang et al. (43) found that in addition to the helicase domain, the zinc-binding domain also plays a vital role in the replication and transcription of coronaviruses. In addition to its role in the IBV replication process, IBV nsp13 can interact with the DNA polymerase of host cells (44), causing damage to the normal replication process of host cells and blocking the cell cycle at S phase or G2/M phase to promote the replication of IBV itself and the production of progeny viruses.

### Nsp13, Nsp14, and Nsp16: Formation of Viral mRNA Cap Structure

The cap structure of viral mRNA plays an extremely important role in the viral replication process. In detail, the cap structure of mRNA can be used as a starting point to promote protein translation, protect mRNA from cellular 5′-3′ exonuclease degradation, and limit the innate immune system to recognizing viral mRNA (45, 46). In recent years, studies have proved that coronaviruses adopt the classic eukaryotic pathway to form its cap-like structure (41).

In addition to the functions described above, the nsp13 is also predicted to have RTPase activity, which can remove 5′-γ-phosphorylation sites on mRNA and participate in the first step of RNA cap structure formation (41).

As we know, coronavirus nsp14 contains 3′-5′ exoribonuclease (ExoN) and guanine-N7-methyltransferase (N7-MTase) domains (47). The former is predicted to provide proofreading activity, which is essential for viral RNA synthesis with high replication fidelity (48, 49). Additionally, SARS-CoV nsp14 was identified as a N7-MTase at first (50) and then it was found that the nsp14 N7-MTase domains of coronaviruses are highly conserved in genome motifs, including gamma-coronavirus IBV (47). So, it can be predicted that IBV nsp14 also has a N7-MTase activity, which can participate in the formation of viral mRNA cap structure and methylate guanine at the N7 site to produce the so-called “cap 0” structure (41).

Nsp16 has been confirmed to have 2′-O-methyltransferase activity which can participate in the final step for the formation of the mRNA cap structure, transforming the “cap 0” structure into the “cap 1” structure (41). It makes viral mRNA to exhibit the same structure as eukaryotic mRNA, thereby evading host immune recognition.

### Nsp15: Inhibit the Formation of Cytoplasmic Stress Granules

Nsp15 of coronaviruses was identified as a highly conserved endonuclease with uridine acid specificity (51). When nsp15 was knocked out in IBV genome, a study found that the expression of IBV mRNA and proteins in the chicken embryo kidney cells were both reduced compared with the wild type strain, which indicated that the replication of IBV was severely impaired without nsp15 (52). In addition, it has been proved that IBV nsp15 can inhibit the formation of cytoplasmic stress granules (SGs) by preventing PKR activation (53). Cytoplasmic SGs are usually produced by stress-induced translational block, which are considered as the platforms required for innate immunity initiation and PKR activation (22). Recent studies have shown that SGs can participate in protein synthesis, shutdown, and recruitment of innate immune signal transmission intermediates to exert antiviral functions (52, 53). In summary, IBV nsp15 can inhibit the formation of SGs by antagonizing the PKR activation to achieve immune evasion.

## Accessory Proteins

Among the open reading frame encoding structural proteins, some non-structural proteins are also encoded, called accessory proteins. These accessory proteins are also considered to be involved in regulating virus replication or immune evasion (2).

### 3a and 3b: Inhibit the Formation of Type I Interferon

3a, 3b and envelope proteins are the protein expression products of mRNA3 in the nested transcription mechanism of IBV (9, 54). Initially, it was found that 3a and 3b are located in the smooth endoplasmic reticulum (55) and cell cytoplasm (56), respectively. Subsequently, Shen et al. (57) and Hodgson et al. (58), respectively, proved that 3a and 3b are not necessary for IBV replication, indicating that 3a and 3b are not directly involved in the formation of IBV RNA or proteins. However, an *in vivo* study showed that the deletion of 3a protein in IBV impaired the ability for IBV replication on chicken embryos (59). Another study also found that the deletion of 3a and 3b led to the weakening of the pathogenicity of the QX type IBV strain on chicken embryos, while the effect of 3b protein was greater than that of 3a protein (60). The results of *in vivo* activity experiments alone are insufficient to confirm the specific roles of 3a and 3b in IBV infection. However, recently, there are *in vitro* studies have proved that accessory proteins 3a and 3b can antagonize the host innate immune response triggered by IBV, which provided a new idea for the functions of accessory proteins 3a and 3b. After IBV infection, 3a and 3b can antagonize the host innate immune response, and delay the activation of intracellular type I IFN and IFN stimulating factors (61). The deleted 3a IBV strain has a weakened resistance to type I IFN, indicating that 3a can indeed antagonize IFN activity (62).

### 5a and 5b: Shut Down the Host Protein Synthesis

Similarly, the accessory proteins 5a and 5b were also initially reported to prove that they are not directly involved in the replication process of IBV and not necessary for the replication process (63, 64). In an *in vivo* study, the 5b deficient IBV strain could not induce delayed activation of IFN compared with the wild type strain, which revealed that the 5b protein is involved in the process of IBV antagonizing interferon (65). Zhao et al. (66) compared the genome sequence of IBV virulent strain YN with attenuated strain aYN, and observed cumulative mutations in the 5a gene. Further research found the mortality, tissue damage and virus titers of the 5a deficient IBV strain were all reduced. The result confirmed that the 5a protein is closely related to the pathogenicity of IBV, and the lack of 5a protein is one of the reasons for the attenuated virulence of IBV strains (66). Compared with the wild type strain, the 5b deficient IBV strain can make the host cells produce a higher concentration of type I interferon in an *in vitro* cell environment (67). Kint et al. (67) further confirmed that 5b protein can antagonize the innate immune response, and the mechanism of this immune evasion function may be due to the 5b protein having the function of shutting down host protein synthesis, which is similar to the function of the nsp1 encoded by alpha and beta coronaviruses (68–71). It suggested that the 5b protein of IBV may compensate for the effect of nsp1 protein deletion on IBV virulence.

## Discussion and Perspective

**Figure 2:**
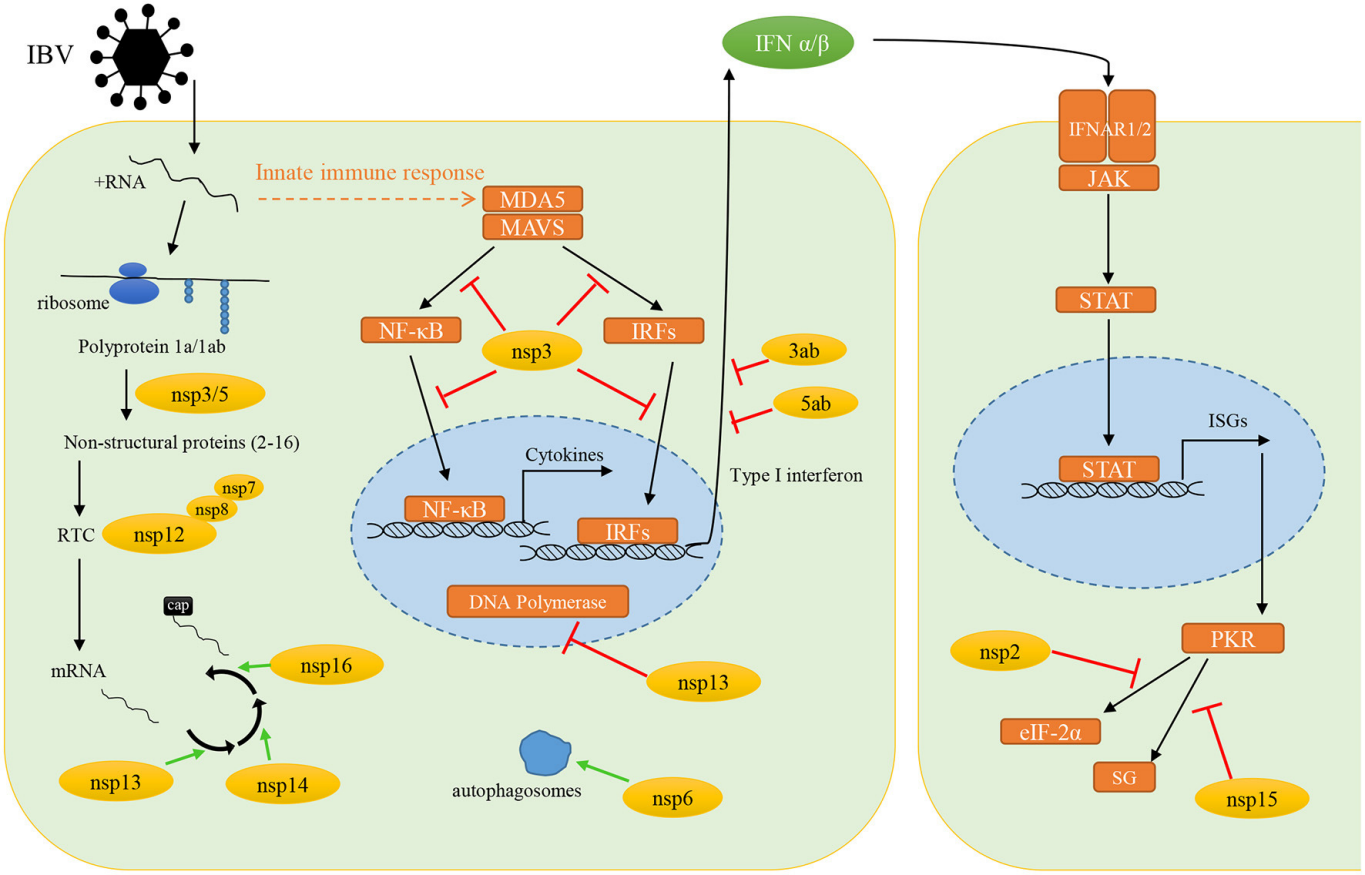
The immune evasion functions of infectious bronchitis virus non-structural and accessory proteins. Yellow circles, IBV non-structural proteins; orange boxes, host proteins; green arrows, promote the processes; red arrows, inhibit the protein function or signal transduction.

When IBV infects cells, it needs to create essential conditions for its own replication in host cells, and at the same time it must evade the surveillance of host innate immune response. To achieve these two conditions, the non-structural proteins encoded by IBV play an indispensable key role in it. In this review, we summarized the current knowledge on immune evasion functions of IBV non-structural and accessory proteins (Figure 2). Such as the nsp3 through its deubiquitinating enzyme activity, which can extensively regulate the host innate immune pathways, and ultimately antagonize the production of type I interferon. The nsp6 can enhance autophagosomes formation to favor its self-assembly, while limiting the size of autophagosomes to compromise the ability of autophagosomes to deliver viral components to lysosomes for degradation. The nsp13, nsp14, and nsp16 are predicted to promote the formation of viral mRNA cap structure to avoid the recognition by host innate immune response. All in all, there is a large number of reports on the immune escape mechanism mediated by IBV non-structural proteins. However, there are still some unknown functions waiting for us to explore. In order to meet the current challenges in the prevention and treatment of IBV, understanding the immune evasion functions of non-structural proteins and their role in the pathogenic mechanism is of great significance for the prevention and treatment of IBV in the future.

## Author Contributions

SP, YW, and YZh: conceptualization. SP: writing—original draft preparation. YW and YZh: writing—review and editing. XS and XZ: visualization. YZo and LL: supervision. ZY: project administration and funding acquisition. All authors have read and agreed to the published version of the manuscript.

## Funding

This research was supported by the Program Sichuan Veterinary Medicine and Drug Innovation Group of China Agricultural Research System (SCCXTD-2020-18) and the Science and Technology Project of Sichuan Province (2021NZZJ0021).

## Conflict of Interest

The authors declare that the research was conducted in the absence of any commercial or financial relationships that could be construed as a potential conflict of interest.

## Publisher's Note

All claims expressed in this article are solely those of the authors and do not necessarily represent those of their affiliated organizations, or those of the publisher, the editors and the reviewers. Any product that may be evaluated in this article, or claim that may be made by its manufacturer, is not guaranteed or endorsed by the publisher.
